# Fibroblast growth factor 21 facilitates peripheral nerve regeneration through suppressing oxidative damage and autophagic cell death

**DOI:** 10.1111/jcmm.13952

**Published:** 2018-11-18

**Authors:** Yingfeng Lu, Rui Li, Junyi Zhu, Yanqing Wu, Duohui Li, Lupeng Dong, Yiyang Li, Xin Wen, Fangzheng Yu, Hongyu Zhang, Xiao Ni, Shenghu Du, Xiaokun Li, Jian Xiao, Jian Wang

**Affiliations:** ^1^ Department of Hand Surgery and Peripheral Neurosurgery The First Affiliated Hospital of Wenzhou Medical University Wenzhou Zhejiang China; ^2^ Molecular Pharmacology Research Center School of Pharmaceutical Science Wenzhou Medical University Wenzhou Zhejiang China

**Keywords:** autophagic cell death, fibroblast growth factor 21, oxidative stress, peripheral nerve injury, remyelination

## Abstract

Seeking for effective drugs which are beneficial to facilitating axonal regrowth and elongation after peripheral nerve injury (PNI) has gained extensive attention. Fibroblast growth factor 21 (FGF21) is a metabolic factor that regulates blood glucose and lipid homeostasis. However, there is little concern for the potential protective effect of FGF21 on nerve regeneration after PNI and revealing related molecular mechanisms. Here, we firstly found that exogenous FGF21 administration remarkably promoted functional and morphologic recovery in a rat model of sciatic crush injury, manifesting as persistently improved motor and sensory function, enhanced axonal remyelination and regrowth and accelerated Schwann cells (SCs) proliferation. Furthermore, local FGF21 application attenuated the excessive activation of oxidative stress, which was accompanied with the activation of nuclear factor erythroid‐2‐related factor 2 (Nrf‐2) transcription and extracellular regulated protein kinases (ERK) phosphorylation. We detected FGF21 also suppressed autophagic cell death in SCs. Additionally, treatment with the ERK inhibitor U0126 or autophagy inhibitor 3‐MA partially abolishes anti‐oxidant effect and reduces SCs death. Taken together, these results indicated that the role of FGF21 in remyelination and nerve regeneration after PNI was probably related to inhibit the excessive activation of ERK/Nrf‐2 signalling‐regulated oxidative stress and autophagy‐induced cell death. Overall, our work suggests that FGF21 administration may provide a new therapy for PNI.

## 1 INTRODUCTION

Peripheral nerve injury (PNI) is a common chronic disease in clinical practice due to accidental trauma, surgery or inflammatory disorders. Moreover, such injuries affect more than 20 million people annually in the United States alone.^1^ Damage to the peripheral nerves may result in motor and sensory dysfunction and irreversible tissue atrophy.^2^, ^3^ Although the adult mammalian peripheral nervous system has a considerable potential for spontaneous recovery, the complex and chronic recovery process often leads to unsatisfactory repair results.^4^ At present, direct surgical repair, nerve grafting and nerve bridging are still regarded as the golden standard therapeutic approaches for peripheral nerve rehabilitation.^5^ However, those surgical operations have several disadvantages, including need for a second operation, insufficient nerve donors and possible formation of neurofibroma around the focal area.^2^, ^4^ Therefore, seeking novel drug therapy to overcome these shortcomings has become very essential.^6^, ^7^

Molecular therapy utilizing fibroblast growth factors (FGFs) to promote peripheral nerve myelination and regrowth has been used almost universally.^8^ FGFs are a family of cell signalling molecules involved in comprehensive biological properties and participate in regulating cell differentiation, proliferation and metabolism.^9^, ^10^ As one member of those family, fibroblast growth factor 21 (FGF21) exerts pleiotropic effects on modulating glucose, lipid and energy homeostasis.^11^, ^12^ It is mainly secreted by the liver, pancreas, kidney, skeletal muscle and fat.^13^ Studies on obese rodents have demonstrated that administration of recombinant FGF21 significantly reduced hyperglycemia and hepatic triglyceride concentration, improved energy expenditure and alleviated insulin resistance.^14^, ^15^ Apart from its metabolic regulation, recently, there is increasing evidence indicating that FGF21 possesses protective effects on myocardial ischaemia and neurotrauma.^16^, ^17^ For example, Planavila et al. have identified FGF21 protected the heart from ischaemia‐reperfusion via controlling oxidative stress signalling.^18^ FGF21 is also a key mediator for glutamate‐induced excitotoxicity and D‐galactose‐induced ageing in the central nervous system (CNS).^19^, ^20^ Furthermore, it is reported that circulating FGF21 levels increased the injury lesion to promote remyelination after traumatic brain injury.^21^ However, little work has been done to explore FGF21 on nerve regeneration after PNI and clarify its underlying mechanisms.

Oxidative stress is a highly disordered metabolic process that generates the imbalance between oxides and antioxidants.^22^ Peripheral nerves are susceptible to oxidative stress, and it has been reported that reactive oxygen species (ROS) production showed a significant increase after PNI.^23^ Once the peripheral nerve suffered damage, the lesion site and its distal stump are accompanied by extensive ischaemic and inflammatory processes, resulting in a redundant accumulation of ROS. At the same time, the levels of endogenous antioxidants, including superoxide dismutase (SOD), NADH dehydrogenase quinone 1 (NQO1) and Heme oxygenase‐1 (HO‐1), are not sufficient to maintain the requirement for resisting oxidative damage in the trauma region, which finally result in cell apoptosis and death.^24^, ^25^, ^26^ In the antioxidant defensive system, Nuclear factor erythroid 2‐related factor 2 (Nrf‐2) as a pivotal transcription factor for regulating oxidative stress, is able to translocate into the nucleus after separating from Kelch‐like ECH‐associated protein 1 (Keap1) in the cytoplasm to initiate transcription of antioxidant genes including NQO1 and HO‐1 to recover cellular normal physiological function.^27^, ^28^ As one of upstream signal for regulating Nrf‐2, extracellular‐regulated protein kinase (ERK) is a serine–threonine kinase that participates in the modulation of cell proliferation, differentiation and cytoskeletal dynamics.^29^ After PNI, ERK activates the downstream pathway of Nrf‐2 to promote SC proliferation and nerve repair.^30^, ^31^ In addition, the ERK/Nrf‐2 signalling pathway ameliorates diabetic neuropathy induced‐oxidative damage.^32^ Thus, we speculate that ERK/Nrf‐2‐ mediated antioxidative process may be the potential molecular mechanism for regulating FGF21 neuroprotective property.

Autophagy is an intracellular catabolic progression that degrades the certain amounts of waste proteins or damage organelles in double‐membraned vesicles to maintain metabolize homeostasis. During the last decade, autophagy has been found to be strongly connected with nervous system disease.^33^ Generally, moderate autophagy is regarded as the neuroprotective effect. However, an excessive level of autophagy may lead to nonapoptotic cell death, which is known as autophagic cell death.^34^ Various studies have been shown that abnormal autophagy activation aggravated neurotraumatic disease such as ischaemia stroke, traumatic brain injury (TBI) and acute spinal cord injury.^35^, ^36^, ^37^ Using autophagic inhibitor 3‐methyladenine (3‐MA) or knockout autophagy‐related gene could decrease those neurotrauma‐induced cell death.^38^ Shi et al have reported that miR‐195 increased inflammation‐induced neuropathic pain by controlling microglial autophagy activation following PNI.^39^ However, whether the effect of FGF21 on regulating neuroprotection is associated with suppressing autophagic cell death remains to be elucidated.

In this study, we systematically evaluated the role of FGF21 in axonal regeneration and remyelination and revealed its molecular mechanism following clip compression induced PNI. Our results found that exogenous administration of FGF21 enhanced the speed and extent of functional recovery, promoted nerve regrowth and remyelination, and facilitated SC proliferation. Moreover, this neuroprotective effect of FGF21 was related to the inhibition of excessive oxidative stress activation and autophagic cell death. The former response was probably regulated by the ERK/Nrf‐2 signalling pathway. Our findings shed light on an effective and feasible clinical use of FGF21 for PNI repairing.

## 2 MATERIALS AND METHODS

### 2.1 PNI model and drug administration

Adult male Sprague‐Dawley rats (200‐250 g) were obtained from the Animal Center of the Chinese Academy of Science (Shanghai, China). The living conditions and experimental procedures conformed to the National Institutes of Health (NIH) Guide Concerning the Care and Use of Laboratory Animals. All animal experiments described were approved by the Animal Experimentation Ethics Committee of Wenzhou Medical University, Wenzhou, China. Five rats per cage were housed under controlled environmental conditions with regard to temperature (23 ± 2°C), humidity (35%‐60%), and a 12:12 h light–dark cycle.

The PNI model was described previously.^40^ Briefly, after anaesthetizing by 10% chloral hydrate (3.5 mL/kg), all the rats were fixed on the operating table in a prone position. A 1 cm incision was made in the middle of the thigh to expose the right sciatic nerve. The sciatic nerve was then clamped with two vascular clips (30 g force for 2 minutes, Oscar, China) to form moderate crushing injury. One vascular clip was placed at the distance 7 mm proximal to the sciatic nerve trifurcation. Two vascular clips were clipped approximately at 2 mm intervals. Thereafter, the wound was immediately closed with nondegradable sutures. All rats were then randomly divided into two crushed groups: PNI and FGF21, n = 10 for each group. As for the sham group, they received the same surgical procedure without contusing the sciatic nerve. As for FGF21 group, they received 100 μL of 500 μg/mL FGF21 (FGF21 powder was dissolved in PBS. It was produced from the School of Pharmacy, Wenzhou Medical College, Wenzhou, China) via intramuscular injections once daily for seven consecutive days after injury. Both the sham and PNI groups were administrated the same volume of saline. Twenty‐eight days later, all animals were killed, and the right sciatic nerves were collected to assess the pathology index.

### 2.2 Walking track analysis

To evaluate motor function recovery, walking track analysis was performed on all the rats at 1, 2, 3, and 4 weeks postsurgery as previously described.^40^ The trained rats walked through a confined glass box (90 cm × 15 cm × 20 cm) which placed a white paper (90 cm × 18 cm) on the bottom. The walking paw prints were recorded to calculate the sciatic functional index (SFI, proposed by Bain 41). The experimental process was repeated three times by the same investigator who never participated in the behavioural experiments.

### 2.3 Von Frey hair test

Before performing the Von Frey hair test, all experimental rats were habituated in a glass cubicle for at least 1 hour. The bottom of the cubicle was made of wire mesh with a drilled surface. Each drill hole was 5 × 5 mm^2^, and the hole spacing was 1 mm. To assess the tactile threshold, the Von Frey filaments (NC12775; North Coast Medical Inc, CA, USA), with increasing irritation starting at 2 g, were placed perpendicular to the planta pedis and pressed until a perceptible bend of approximately 90° was observed for 6‐8 seconds. Forces that induce dodge reactions including paw withdrawal, delayed fall, jitter, licking reaction or rats’ avoidance were recorded to calculate withdrawal thresholds. The experimental process was repeated three times with an interval of 15 minutes by the same investigator who was blinded to the group design.

### 2.4 Morphological and histological analysis

The collected sciatic nerves containing the area of crush injury site were cut into two parts. One segment (about 1 cm) was fixed in cold 4% paraformaldehyde in 0.01 M phosphate‐buffered saline (PBS, pH 7.4) overnight. After being embedded in paraffin, longitudinal or transverse sections (5 μm thick) were sliced for haematoxylin/eosin (HE) staining according to the manufacturer's instruction. Images were photographed under a light microscope. Another 2 mm segment was immersion fixed and processed by standard procedures.^42^ Sequentially sectioned slides were examined and photographed under a transmission electron microscope (TEM, H‐600, HITACHI, Japan). Image‐Pro Plus software was used to measure myelin thickness (MT) and axonal diameter (AD). Fibre diameter (FD) was calculated according to the formula: 2 × MT + AD. Finally, an optimal structural indicator including axonal myelination, the G‐ratio (AD/FD), was calculated.

### 2.5 Cell culture and treatment

The rat Schwann cell line, RSC96, was obtained from ScienCell Research Laboratories. Cells were maintained in Dulbecco's modified Eagle Medium (DMEM) containing 10% foetal bovine serum (FBS) (Thermo Fisher Scientific) and incubated in a humidified atmosphere containing 5% CO_2_ at 37 °C. After two passages, cells were passaged into a 96‐well plate at a density of 5000 cells/well to perform the Cell Counting Kit‐8 (CCK‐8) experiments. For protein extraction and ROS estimation, cells were seeded on 6‐well plates at an initial density of 2 × 10^5^ cells/mL. For determining the effect of FGF21, RSC96 cells were divided into three groups: (a) the control group, (b) H_2_O_2_‐treated group, and (c) H_2_O_2_ + FGF21‐treated group. Control group was grown in normal DMEM. H_2_O_2_‐stimulating group were cultured in DMEM with 100 μmol L^−1^ H_2_O_2_ for 4 hours. In the H_2_O_2_ + FGF21 treated group, various concentrations (10, 250 and 1000 ng/mL) of FGF21 was added 2 hours prior to the addition of H_2_O_2_. To further evaluate the effect of ERK/Nrf‐2 on oxidative injury or abnormal autophagy on cell death, SCs were pretreated with ERK inhibitor U0126 (20 μmol L^−1^, HY‐12031) before FGF21 treatment or 3‐methyladenine (3‐MA, 2.5 mmol L^−1^, M129496) before H_2_O_2_ treatment for 2 hours.

### 2.6 Cell viability assay

The CCK‐8 (Beyotime Institute of Biotechnology, PR China) method was used to evaluate cell survival. 10 μL of the CCK‐8 solution was added to each well and then incubated for 2 hours at 37°C. The absorbance values were measured by spectrophotometry at 450 nm with a microplate reader (Thermo Fisher Scientific, Waltham, MA, USA). The cell viability (%) was calculated using the following formula:

$$\text{The cell viability}\left(\%\right)=\left(A_{\mathrm{examination}}-A_{\mathrm{blank}}\right)/\left(A_{\mathrm{control}}-A_{\mathrm{blank}}\right)\times 100\%,$$

where A_examination_ and A_control_ represented the measuring optical density (OD) for drug treating samples and for controlling cells, respectively. A_blank_ was the OD value of wells without cells. The assays were repeated three times.

### 2.7 Determination of intracellular ROS generation

Intracellular ROS levers were detected using the Reactive Oxygen Species Assay Kit (S0033, Beyotime, China) according to the manufacturer's instructions. Briefly, after adding H_2_O_2_ with/without FGF21, an appropriate volume of diluted 2′, 7′‐dichlorodihydrofluorescein diacetate (DCFH‐DA) was added in the dusk of the room and incubated at 37°C for 20 minutes in the incubator. Thereafter, cells were removed from the flow tube. The fluorescence intensity of 2′, 7′‐dichlorofluorescein (DCF) was detected by FACScan flow cytometry (Becton, Dickinson and Company, Franklin Lakes, NJ, USA). For each sample, 10 000 cells were collected.

### 2.8 Immunofluorescence staining

The immunofluorescent process of tissue or cells was done as previously described.^40^, ^43^ The detailed information of primary and secondary antibodies was shown as following: NF‐200 (1:10000, Abcam, ab4680), MBP (1:200, Abcam, ab40390), HO‐1 (1:100, Santa Cruz, sc‐1797), NQO1 (1:1000, Abcam, ab34173), S100 (1:200, Abcam, ab4066), Nrf‐2 (1:500, Abcam, ab62352), Alexa‐Fluor 488 donkey anti‐ rabbit IgG (1:1000, Abcam, ab150073), and Alexa‐Fluor 594 donkey anti‐mouse IgG (1:1000, Abcam, ab150108). Nuclei were labelled with 4′6‐Diamidino‐2‐phenylindole‐dihydrochloride (DAPI, C1006; Beyotime Institute of Biotechnology, Shanghai, China). All fluorescence images were obtained under the Nikon ECLIPSE 80i (Nikon, Tokyo, Japan).

### 2.9 Western blotting analysis

Total proteins were extracted using RIPA buffer containing protease and phosphatase inhibitors for the surgical sciatic nerves or RSC96 cells. The protein concentration was quantitatively analysed, using a BCA protein assay kit (Thermo Fisher Scientific, Rockford, IL, USA). Samples containing 80 μg total protein were separated from 10% or 12% SDS‐polyacrylamide gels and transferred into the nitrocellulose membrane PVDF (Bio‐Rad, Hercules, CA, USA). After blocking with 5% nonfat milk, the membranes were incubated with the following primary antibodies: GFAP (1:10000; Abcam, ab10062), S100 (1:200, Abcam, ab4066), MBP (1:1000; Abcam, ab40390), ERK (1:100; Santa Cruz, sc‐14), *p*‐ERK (1:1000; Cell Signaling Technology, 4370S), Nrf‐2 (1:1000; Abcam, ab62352), HO‐1 (1:100; Santa Cruz, sc‐1797), NQO1 (1:1000; Abcam, ab34173), Bcl‐2 (1:1000; Abcam, ab59348), BAX (1:1000; Cell Signaling Technology, 14796S), Actived‐caspase3 (1:1000; Abcam, ab2302), Beclin‐1 (1:2000; Abcam, B6186), Atg‐5 (1:1000; Cell Signaling Technology, A074), and LC3 (1:50; Novus, NB100‐2220) overnight at 4°C. Then, the membranes were washed with TBST three times and incubated with horseradish peroxidase‐conjugated secondary antibodies (1: 10000) for 1 hour. GAPDH (1:10000; MultiSciences, 70‐Mab5465‐100) was used as an internal control. Signals were visualized and band density was quantified, using a ChemiDocXRS + imaging system (Bio‐Rad Laboratories, Hercules, CA, USA). The above experiment was repeated three times.

### 2.10 Statistical analysis

Statistical analysis was performed using GraphPad Prism 5.0 (GraphPad Software Inc., La Jolla, CA, USA). The results are plotted as the means ± SEM. One‐way ANOVA followed by Tukey's test was used for walking track analysis, von Frey hairs test, immunoblotting and immunofluorescent evaluation. For all comparisons, statistical significance was defined as *P* < 0.05.

## 3 RESULT

### 3.1 FGF21 promotes motor and sensory functional recovery after PNI

**Figure 1:**
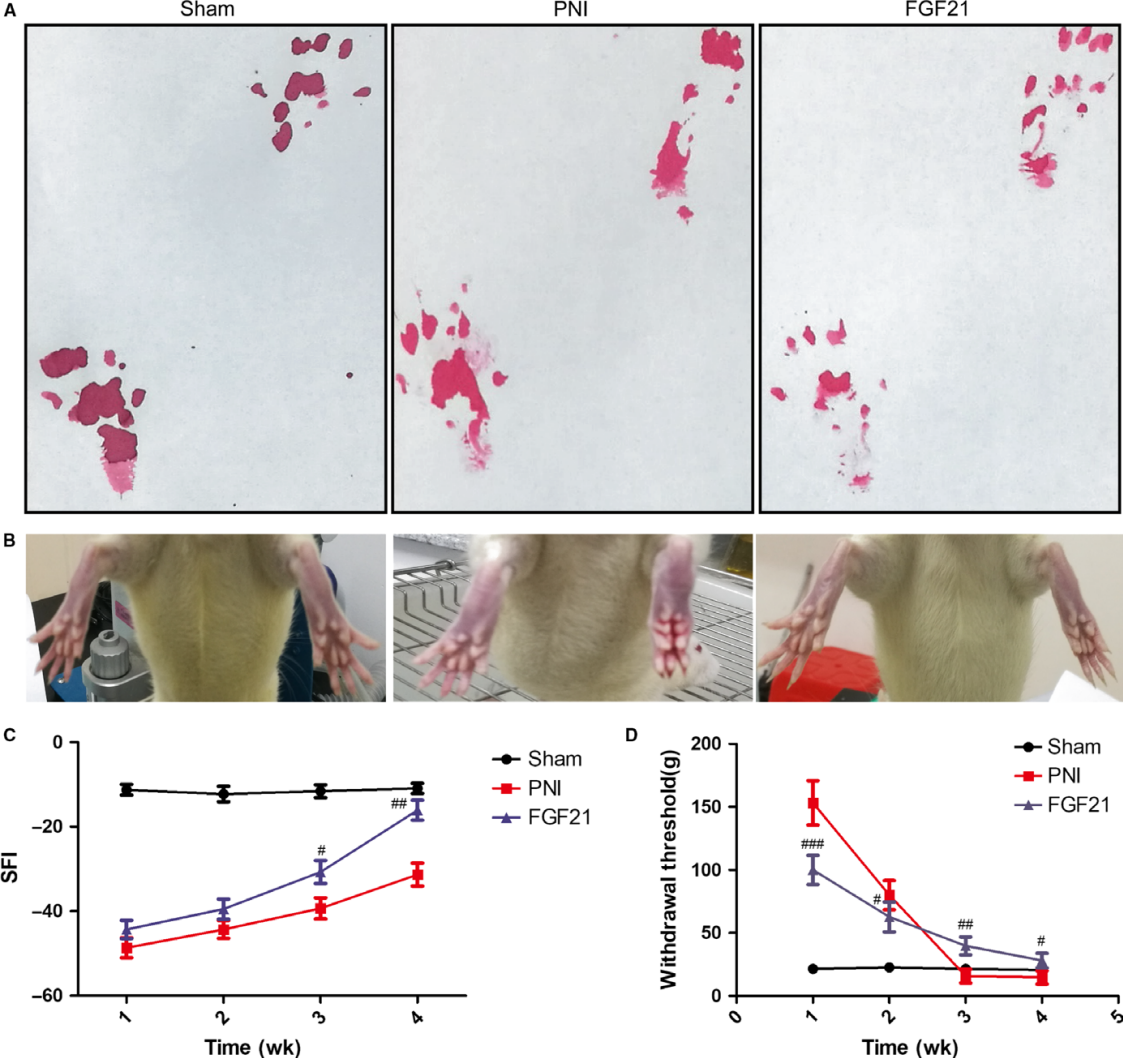
FGF21 promotes locomotor and sensory recovery after PNI. A, Pictures of the rat walking track prints in each group at 4 weeks after sciatic nerve crush. B, Representative photographs of rat hindlimb at 4 weeks recovery following drug treatment. C, Sciatic function index (SFI) analysis in each group at 1, 2, 3 and 4 weeks. D, Withdrawal threshold measured by von Frey hairs test at indicated time points. ^#^ *P* < 0.05, ^##^ *P* < 0.01 and ^###^ *P* < 0.001 versus the PNI group. All these data were represented as the means ± SEM, n = 10

To evaluate whether exogenous FGF21 administration could have a therapeutic role on the PNI model, we persistently injected 50 μg FGF21 solution into the right injured hind limb for 1 week. The locomotor and sensory recovery throughout the 4‐week period was evaluated, using the walking track analysis and the von Frey test. The SFI progressively improved with time but detected no differences across the two crushed groups before week 2. At 3 weeks after treatment, the SFI value of the FGF21 group was superior to PNI group, but inferior to the sham group. This different became even more remarkable at week 4 (Figure 1A, C, ^##^ *P* < 0.01). The plantar views of the hind paws spreading was also showed the same trend (Figure 1B).

We next measured the mechanical allodynia of the right hind limb in these three experimental groups at 1‐4 weeks using the Von Frey test. The results demonstrated that FGF21‐treated rats had a high mechanical sensitivity than the PNI group in the first 2 weeks after injury. In contrast, the withdrawal threshold in the FGF21 group was significantly reversed at 3 and 4 weeks postinjury (Figure 1D, ^#^ *P* < 0.05), suggesting that the FGF21 treating rats could avoid to provoke hyperpathia at the later stage of nerve recovery. Overall, these observations suggest that exogenous administration of FGF21 is beneficial to improving the recovery of both motor and sensory function after crush injury.

### 3.2 FGF21 enhances myelinated fibres regeneration after PNI

**Figure 2:**
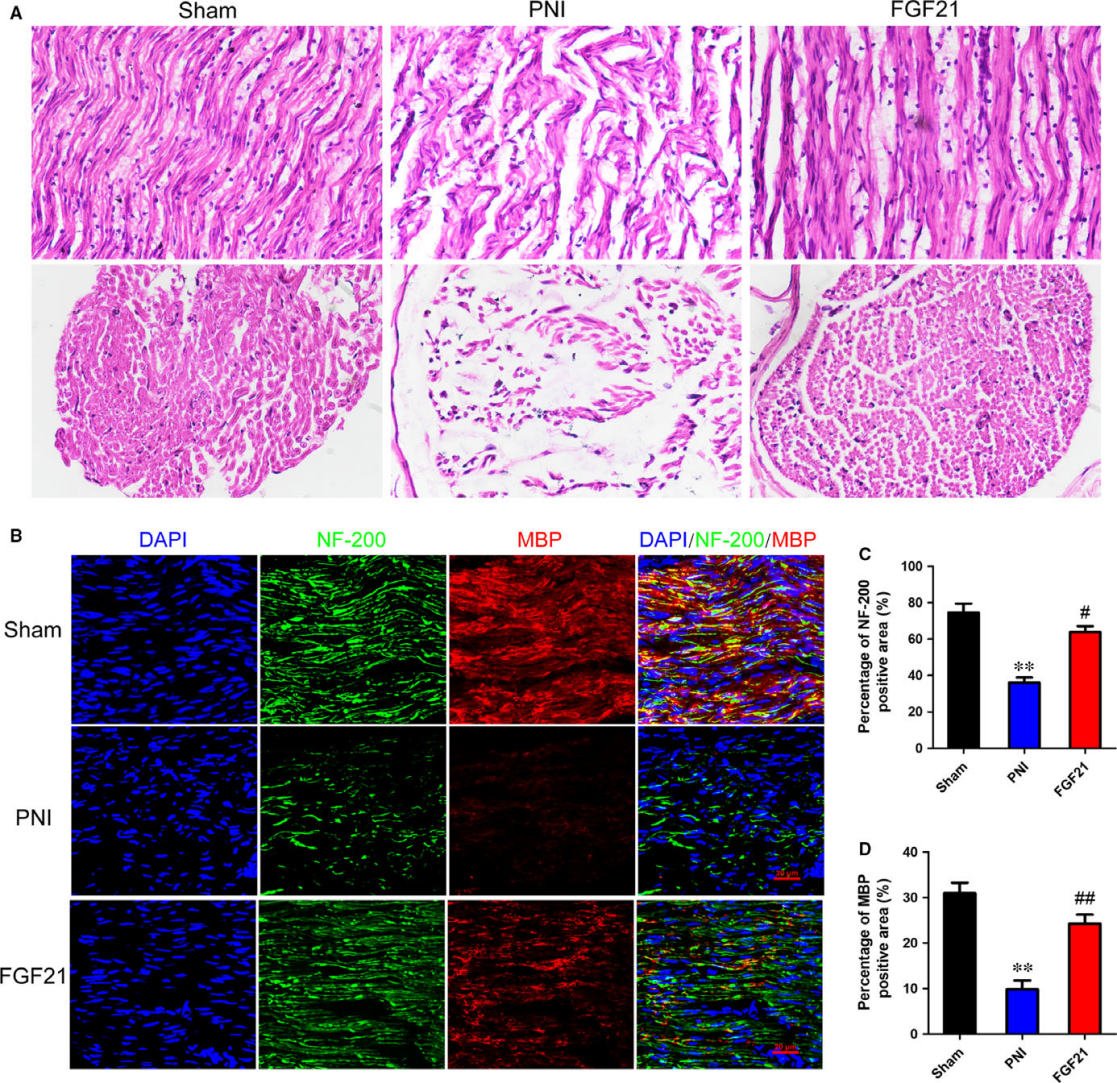
FGF21 promotes myelinated nerve fibers regeneration. A, H&E staining of sciatic nerve. Light micrographs of transverse or longitudinal sections in each group. Scale bar: 40 μm. B, Representative images exhibiting double‐immunofluorescent staining of NF‐200 (green) and MBP (red) of longitudinal sections from the tissue at 28 days after PNI (Scale bar = 50 μm). C and D, Statistical analysis of the NF‐200 and MBP positive staining areas in each group. **P < 0.01 vs the sham group, ^#^ *P* < 0.05 and ^##^ *P* < 0.01 vs the PNI group. All these data represent the means ± SEM, n = 3

After the sciatic nerve injury, axon regeneration is the pivotal step for nerve restore.^44^, ^45^ To evaluate the histologic changes of the injured nerve fibres, H&E staining was performed on longitudinal and transverse sections in all groups after 4 weeks restoration. The results showed that the regenerated nerves in the FGF21 group had denser newborn nerve fibres with compact and uniform style (Figure 2A). Nevertheless, the regenerated nerve fibres in the PNI group were smaller and irregular compared to the FGF21 group. To further confirm the effect of FGF21 on myelinated nerve regeneration, we performed co‐immunofluorescent staining of NF‐200 (a heavy subunit of neurofilaments marking for both large and small axons) and MBP (myelin basic protein, tracking for myelination) on longitudinal sections. The results are shown in Figure 2b, the density of myelinated nerve fibres and axons in the FGF21 group was significantly higher than those in the PNI group. Moreover, statistical analysis of NF‐200 and MBP expression also showed the similar trend (Figure 2C,D, ^#^ *P *< 0.05). All these analyses suggest that FGF21 administration contributes in the accelerating the regeneration of myelinated nerve fibres.

### 3.3 FGF21 promotes remyelination and SCs proliferation after PNI

**Figure 3:**
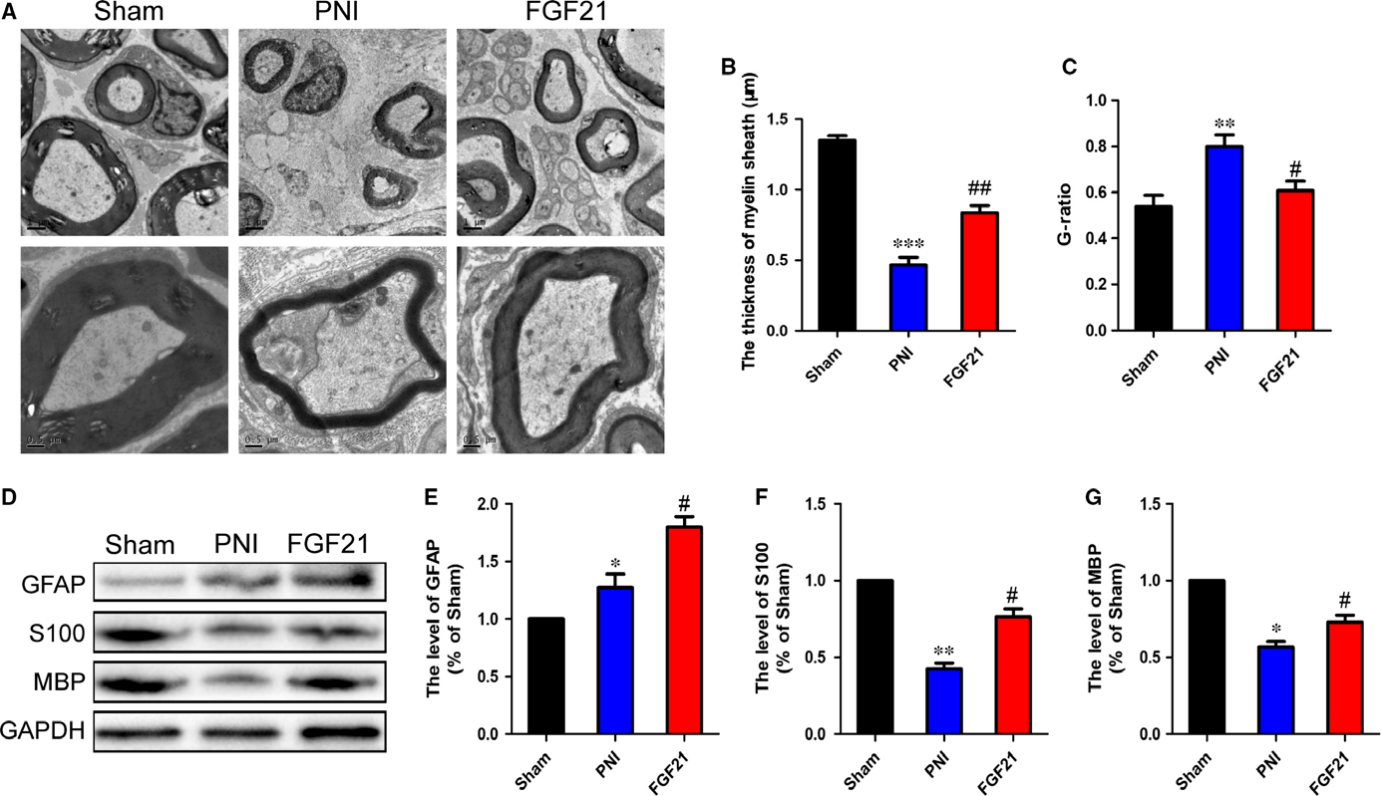
FGF21 promotes sciatic nerve remyelination and SCs proliferation after PNI. A, Representative electron micrographs of sciatic nerve transverse sections from each group. B and C, Statistical analysis of the thickness of myelin sheaths and G‐ratio using the Image‐Pro Plus software. D‐G, Representative western blotting and data analysis of GFAP, S100 and MBP in all groups. *P < 0.05, **P < 0.01 and ***P < 0.001 vs the sham group, ^#^ *P* < 0.05 and ^##^ *P* < 0.01 vs the PNI group. All these data represent the means ± SEM, n = 3

Schwann cells (SCs), the myelin‐ensheathing cell of the peripheral nervous system, are essential for forming myelin the sheath and maintaining myelin thickness.^46^, ^47^ To explore whether the effect of FGF21 on remyelination after PNI was associated with SCs proliferation, we firstly observed the histological changes of the regenerated myelin sheath in each group through transmission electron microscopy (TEM). As shown in Figure 3A, the PNI group exhibited decreased thickness and scattered density of myelin sheaths. Nevertheless, these abnormalities were obviously ameliorated after FGF21 treatment. Meanwhile, the statistical analysis of both myelin thickness and G‐ratio in the FGF21 group was remarkably greater than those in the PNI group (Figure 3B,C, ^#^ *P* < 0.05). We next examined the SCs proliferation via western blotting. Glial fibrillary acid protein (GFAP) is a marker of dedifferentiated or proliferative SCs.^48^ S‐100 as well as MBP are major extrinsic membrane proteins secreted by SCs.^49^ They are the main composition in mature and functional SCs. The results showed that there was an increased GFAP positive expression after nerve injury, while stronger in the FGF21 group. The S‐100 and MBP production were significantly reduced in the PNI group. However, these circumstances were significantly reversed after localized FGF21 treatment (Figure 3D‐G, ^#^ *P* < 0.05). Taken together, all of those results suggest that FGF21 can facilitate the proliferation of SCs after PNI, which is beneficial to remyelination.

### 3.4 FGF21 exerts antioxidative effect in SCs after PNI through activating ERK/Nrf‐2 signalling

**Figure 4:**
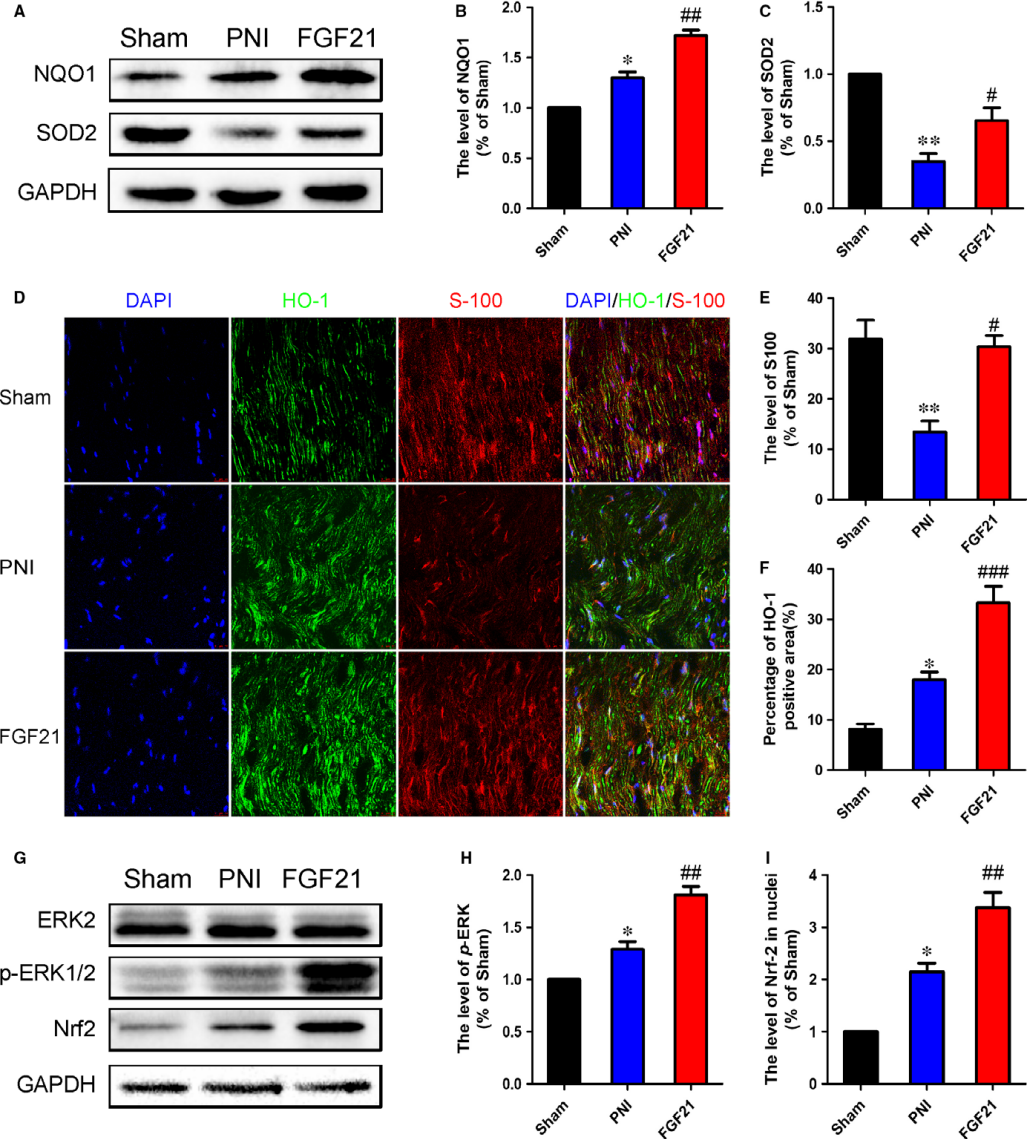
FGF21 upregulates the expression of antioxidant proteins to promote nerve regeneration after PNI. A‐C, Western blotting and quantification data of NQO1 and HO‐1 in each group. D, Representative micrographs showing double immunofluorescence with HO‐1 (green) and S100 (red), nuclei were labelled with DAPI (blue) in each group (Scale bar = 50 μm). E and F, Statistical analysis of the HO‐1 and S100 positive staining areas in each group. G, Western blotting of ERK,* p‐* ERK and Nrf‐2 in each group. H and I, The optical density analysis of *p*‐ERK/ERK and Nrf‐2. *P < 0.05 and **P < 0.01 vs the sham group, ^#^ *P* < 0.05, ^##^ *P* < 0.01 and ^###^ *P* < 0.001 vs the PNI group. All these data represent the means ± SEM, n = 3

As previously described, activation of oxidative stress may cause detrimental effects on the process of remyelination after PNI.^22^, ^50^ To determine whether FGF21 protects injured nerves from damaged by increasing the antioxidant response, the protein expressing levels of NQO1 and HO‐1 were detected through western blotting. The results showed that the lever of NQO1 and HO‐1 were slightly increased after PNI when compared with the control group. However, this trend was evidently up‐regulation after administration of FGF21 (Figure 4A‐C, ^##^ *P* < 0.01). Consistent with the results of western blotting analysis, co‐immunofluorescence staining of HO‐1 and S‐100 showed that FGF21 enhanced HO‐1 expression and reversed the decrease of S‐100 after nerve injury. Notably, HO‐1 was nearly co‐localized with S‐100 in all groups, demonstrating FGF21 enhanced the antioxidative capacity is occurred in SCs (Figure 4D‐F, ^#^ *P* < 0.05).

It is noteworthy that ERK‐suppressing excessive oxidative stress is regulated through Nrf‐2.^51^ To further determine whether FGF21 protected the injured nerves from oxidative damage was through ERK/Nrf‐2 signalling pathway, the levers of Nrf‐2, *p*‐ERK and ERK were measured by western blotting. The results showed the Nrf‐2 expression and the ratio of *p*‐ERK/ERK were increased following PNI and their levels were further enhanced after treating FGF21 (Figure 4G‐I, ^#^ *P* < 0.05). Thus, we propose that FGF21‐exerting antioxidant effect is involved in the ERK/Nrf‐2 signalling pathway.

### 3.5 FGF21 suppresses autophagy‐induced cell death after PNI

**Figure 5:**
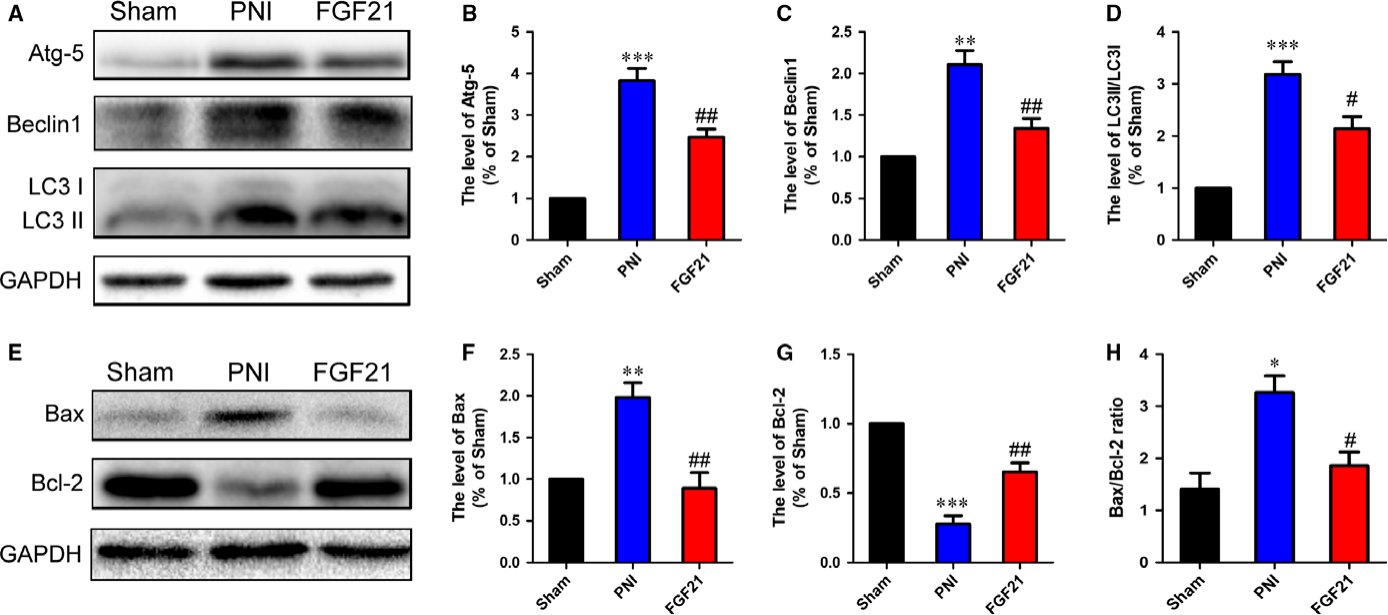
FGF21 administration suppresses autophagy‐induced cell death in vivo. A‐D, Western blotting and quantification data of Atg‐5, Beclin1 and LC3 II/I in each group. E‐G, Western blotting and quantification data of Bax and Bcl‐2 in each group. H, Quantification of Bax/Bcl‐2 ratio in all groups. *P < 0.05, **P < 0.01 and ***P < 0.001 vs the sham group, ^#^ *P* < 0.05 and ^##^ *P* < 0.01 vs the PNI group. All these data represent the means ± SEM, n = 3

Excessive or prolonged autophagy may lead to neuronal cell death.^52^ To demonstrate FGF21 facilitating injured nerve regeneration is also related to attenuate autophagic cell death, we firstly detected the alteration of autophagic biomarkers including Atg‐5, Beclin‐1 and LC3 I/II in each group via western blotting. The results revealed PNI significantly increased the expression of those autophagy related proteins, while FGF21 treatment remarkably decreased this trend (Figure 5A‐D, ^#^ *P* < 0.05). Next, we further determine whether FGF21 treatment is helpful to cell survival. Cell death‐associated proteins, including Bcl‐2 and Bax, were measured by western blotting. The results showed that inhibiting excessive activation of autophagy by FGF21 treatment significantly decreased the level of pro‐apoptotic protein Bax and increased the level of anti‐apoptotic protein Bcl‐2 when compared to PNI group (Figure 5E‐G, ^#^ *P* < 0.05). Statistical analysis also exhibited the ratio of Bax/Bcl‐2 was markedly reduced in FGF21 group when compared to the PNI group (Figure 5H, ^#^ *P* < 0.05). These results suggest that FGF21 plays a crucial role on cell survival by inhibiting excessive autophagy activation.

### 3.6 FGF21 up‐regulates antioxidative ability to improve SCs survival via ERK/Nrf‐2 signalling in vitro

**Figure 6:**
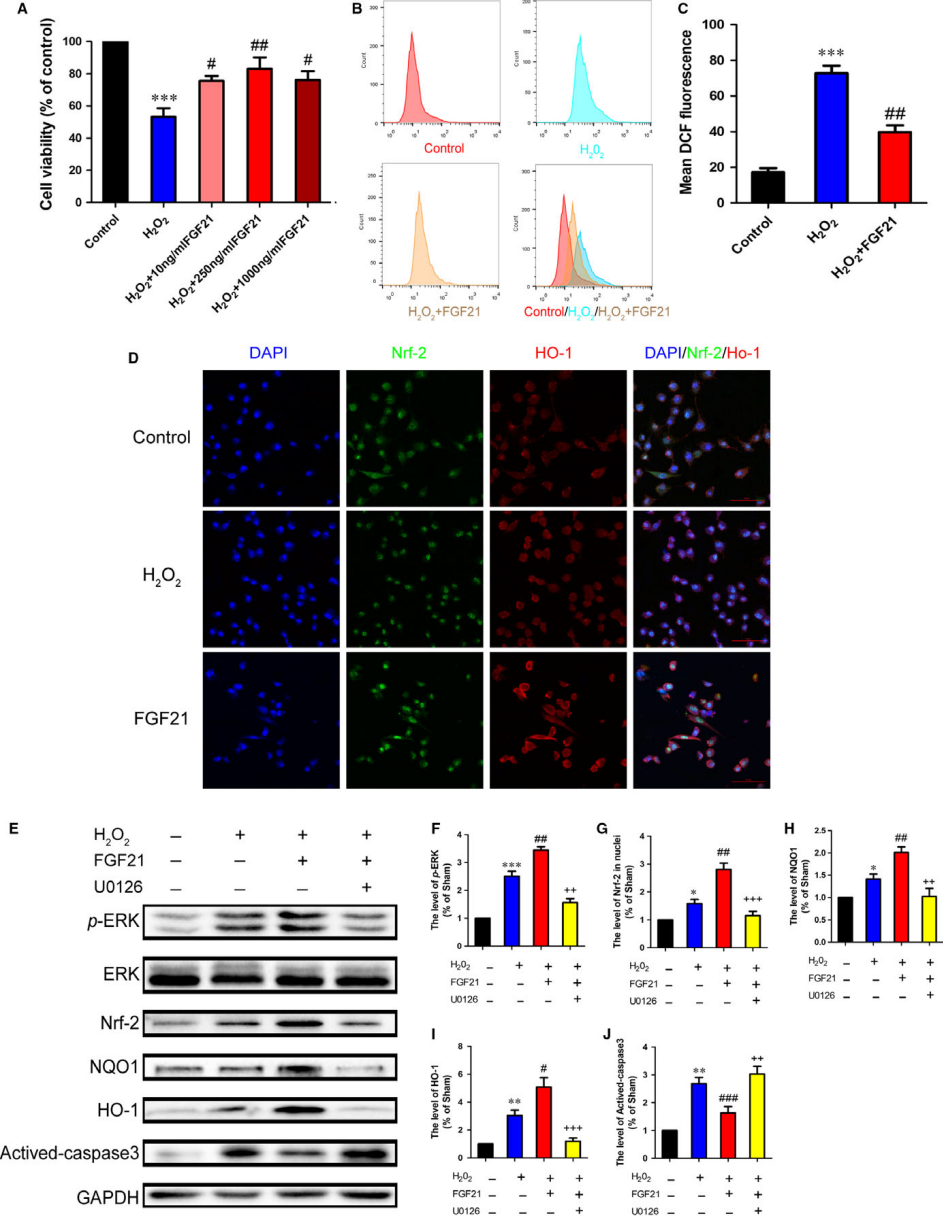
FGF21 promotes the capacity of antioxidant in vitro through ERK/Nrf‐2 signalling. RSC96 cells were pretreated with FGF21 solution before adding H_2_O_2_ (100 μmol L^−1^) in vitro. A, CCK‐8 test was performed to evaluate the number of surviving cells after H_2_O_2_ stimulation. B, RSC96 cells were labelled with DCFH‐DA probes to evaluate the level of intracellular ROS, which was detected by the flow cytometer. C, Quantitative analysis of ROS generation in control, H_2_O_2_ and H_2_O_2_ + FGF21 groups. D, Co‐immunofluorescent images showing the relevance of Nrf‐2 (green) and HO‐1(red) in each group (Scale bar = 50 μm). E‐K, Western blotting and quantification data of *p*‐ERK/ERK, Nrf‐2, NQO1, HO‐1 and Actived‐caspase3 in each group. *P < 0.05, **P < 0.01 and ***P < 0.001 vs the control group, ^#^ *P* < 0.05, ^##^ *P* < 0.01and ^###^ *P* < 0.001 vs the H_2_O_2_ group, ^++^ *P* < 0.01 and ^+++^ *P* < 0.001 vs the H_2_O_2_ + FGF21‐treated group. All these data represent the means ± SEM, n = 3

To investigate the effect of FGF21 on SCs survival, RSC 96 cells, a specialized cloned cell line of SCs, were exposed to H_2_O_2_ (100 μmol L^−1^) for 4 hours to imitate the condition of oxidative damage in PNI in vitro. Meanwhile, those SCs were pretreated with different concentrations of FGF21. As shown in CCK‐8 analysis (Figure 6A), we observed a large number of cell death after H_2_O_2_ stimulation. Pretreatment with FGF21 evidently reversed this circumstance. In addition, FGF21 of 250 ng/mL was regarded as the optimum concentration for improving cell viability. Thus, this concentration of FGF21 was selected for the following experiments.

To determine whether FGF21 decreases the level of intracellular ROS following H_2_O_2_ stimulation in vitro, a special probe for hydrogen peroxide, 2′, 7′‐dichlorodihydrofluorescein diacetate (DCFH‐DA) was added to the cultured cell for analysis of the ROS intensity accumulation by flow cytometer. The results showed that a marked increase of ROS was produced in the H_2_O_2_ group. Nevertheless, pretreated with FGF21 significantly decreased this superlative production of ROS (Figure 6B,C, ^##^ *P* < 0.01). These analyses indicate that FGF21 has a remarkable effect on resisting ROS overgeneration.

Following, the antioxidant level in all groups was detected by double‐immunofluorescence staining of Nrf‐2 and HO‐1. The result revealed that H_2_O_2_ group enhanced the fluorescence intensity of Nrf‐2 in nuclei and HO‐1 in cytoplasm, when compared with control group, those fluorescence intensities were remarkly higher in the FGF21 group (Figure 6D). To further clarify the underlying antioxidant mechanism of FGF21, we hypothesized ERK/Nrf‐2 signalling as the mainly upstream regulator. To test this hypothesis, we used the U0126 to treat SCs with FGF21. Similarly, the ratio of *p*‐ERK/ERK and the levers of Nrf‐2, NOQ1 and HO‐1 expression were slightly upregulation in the H_2_O_2_ group, while this trend was significantly increased when pretreating with FGF21. However, this notably increased levels of *p*‐ERK, Nrf‐2, NOQ1 and HO‐1 by FGF21 treatment, which were reversed by adding ERK inhibitor U0126 (Figure 6E‐I, ^#^ *P* < 0.05). Furthermore, FGF21+ U0126 combination evidently enhanced the expression of Activated‐caspase3 (Figure 6J). Taken together, these results suggest that the neuroprotective of FGF21 for facilitating antioxidant effect to suppress SCs apoptosis is probably through activating the ERK/Nrf‐2 signalling pathway.

### 3.7 FGF21 suppresses autophagy to diminish SCs death in vitro

**Figure 7:**
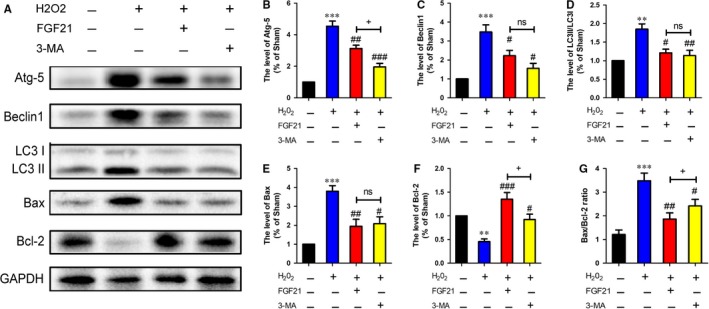
FGF21 plays a protective role in RSC96 cells by suppressing excessive autophagy in vitro. A, Representative western blotting of autophagy and cell death‐associated proteins including Atg‐5, Beclin1, LC3 II/I, Bax and Bcl‐2. B‐F. Quantification of western blotting from A. G, Quantification of Bax/Bcl‐2 ratio in all groups. **P < 0.01 and ***P < 0.001 vs the control group, ^#^ *P* < 0.05, ^##^ *P* < 0.01 and ^###^ *P* < 0.001 vs the H_2_O_2_ group, ^+^ *P* < 0.05 vs the FGF21‐treated group. All these data represent the means ± SEM, n = 3

To further confirm the role of FGF21 in the autophagy‐induced cell death in vitro. Firstly, we detected autophagy‐associated proteins including Atg‐5, Beclin‐1 and LC3 II/I by western blotting. Compared with the control group, significantly higher expression of Atg5 and Beclin‐1, as well as the ratio of LC3 II/I, was seen in the H_2_O_2_ group, while the trends of those autophagy‐associated proteins were significantly reversed when pretreatment with FGF21 or autophagy inhibitor 3‐MA (Figure 7A‐D, ^#^ *P* < 0.05).

We next detected whether FGF21 affected the expression of cell death‐associated proteins in SCs after exposure to H_2_O_2_. Consistent with the results above, western blotting showed that the level of proapoptotic protein Bax was downregulated, while antiapoptotic protein Bcl‐2 was upregulated, and Bax/Bcl‐2 ratio was significantly reduced in FGF21 group when compared to the H_2_O_2_ group. These results were consistent with the 3‐MA‐treated group (Figure 7A, E‐G, ^#^ *P* < 0.05). All of these results demonstrate that FGF21 suppressing SCs death under oxidative stress is partially involved in the inhibition of excessive autophagy.

## 4 DISCUSSION

FGF21 is generally regarded as a potent metabolic regulator.^20^ Larger numbers of researches are focus on its specific effects on controlling glucose and lipid homeostasis, energy metabolism, and insulin sensitivity.^53^ However, in the present study, we are mainly concerned about the effect of FGF21 on mediating neuroprotection and neuranagenesis that tries to reveal its underlying molecular mechanism following PNI. These new discoveries of FGF21‐regulating PNI are concluded as follows: (I) FGF21 is capable of strong neuroprotective effect on the peripheral nervous system (PNS), manifesting in improving functional recovery, promoting both axonal elongation and remyelination, and enhancing SCs proliferation; (II) this neuroprotective of FGF21 is likely associated with enhancing anti‐oxidative capable in SCs in the lesion region to preventing excessive ROS production, which is further regulated by ERK/Nrf‐2 signalling; (III) blocking autophagic cell death is also another effective pathway for FGF21 exerting neuroprotective and neurodevelopmental effect after PNI.

As is known, axonal regrowth and remyelination after PNI is critical for reconstructing a functional nerve that can dominate their appropriate target tissues.^4^ GFs are implicated as the potential therapeutic agents on neurotraumatic disease because of their multiple roles on triggering neuranagenesis, neurotrophy and angiogenesis. In PNS, the synthesis and secretion of GFs primarily come from SCs. However, those endogenous GFs could not satisfy the demands for nerve regeneration. Therefore, supplying exogenous GFs in the injured area becomes necessary for nerve repairing. Previously studied have reported that acid fibroblast growth factor (aFGF), basic fibroblast growth factor (bFGF) and nerve growth factor (NGF) could promote Schwann cell (SC) survival and axonal outgrowth in vivo and in vitro.^54^, ^55^ Part of them have been widely used in clinical applications.^56^ FGF21 is a recently discovered hormone that also belongs to GFs family. Previous researches have confirmed FGF21 is a powerful metabolic regulator for coordinating carbohydrate and lipid metabolism.^57^ But recent studies are involved in its neuroprotection. For example, Chen et al. discovered that FGF21 could decrease the Blood‐brain barrier (BBB) disruption and neuron apoptosis, improve neurofunctional behaviour after traumatic brain injury (TBI).^17^ Kuroda, M group found that peripherally derived FGF21 was contributed to oligodendrocyte proliferation and remyelination when the CNS suffers from pathological damage.^21^ Furthermore, exogenous FGF‐21 protein could absolutely avoid ageing neurons to suffer glutamate‐induced excitotoxicity.^20^ However, it remains unknown that whether these neuroprotective effects of FGF21 on CNS are also potentially acted on pathological PNS disease.

In this study, for the first time, we explored FGF21 on peripheral nerve regeneration through exogenously administrating FGF21 in rat sciatic nerve crush model. We found that FGF21 treatment significantly improved both the speed and extent of recovery of motor and sensory function, as analysed by SFI and mechanical withdrawal response (Figure 1). Moreover, we similarly observed FGF21 treatment remarkably ameliorated histological and morphological recovery through H&E and double‐immunofluorescence staining, manifesting in newborn nerve fibers increasing and regenerative axon growing (Figure 2). It has been reported that SCs proliferation is contributed to remyelination.^48^, ^58^ To address this fact, we examined the expression of SCs‐associated proliferative proteins and the status of myelin remodeling through western blotting and TEM. The results showed that administration of FGF21 was beneficial for the expression of GFAP, S‐100 and MBP, as well as increasing the myelin diameter and thickness, when compared with the PNI group (Figure 3). All of these analyses indicated that FGF21 could exert direct and potent neuroprotective and neurotrophic effects to improve the restoration of neural architecture and function after PNI. But the potential mechanism of FGF21‐regulating peripheral nerve rehabilitation remains largely unknown.

Previous studies showed that oxidative stress was identified to be responsible for the neuronal disease, including PNI and diabetic neuropathy.^59^, ^60^ Kaya et al verified melatonin improved axonal regeneration and functional recovery through reducing oxidative stress in the transected model of PNI.^61^ Moreover, excessive oxidative activation formative superfluous reactive oxygen species (ROS) accumulation leads to neuronal cell apoptosis, which is not beneficial to nerve regeneration.^60^, ^62^ Preventing excessive oxidative stress activation improves the structural and functional recovery after PNI.^22^ Based on the above fact, we speculated inhibiting oxidative stress might be involved in FGF21 ameliorating PNI recovery. To validate this hypothesis, we exposed SCs to H_2_O_2_ to elicit oxidative stress and pretreated it with 250 ng/mL FGF21 with or without a specific inhibitor U0126 in vitro. The results showed massive accumulation of ROS were generated in SCs, which led to a significant reduction of cell viability. But these side‐effects were largely ameliorated with the pretreatment of FGF21 (Figure 6). Consistently, we also observed adding FGF21 markedly enhanced the anti‐oxidative capable in vivo and in vitro, manifesting in obvious increase levers of NOQ1 and HO‐1, which were partially reversed by U0126 (Figures 4 and 6). Furthermore, we detected HO‐1 was nearly co‐localized with S‐100 in all groups, meaning the changes of oxidative stress occurred in SCs. Collectively, all of these results indicate that the depressed antioxidative capacity in SCs after PNI leads to the massive accumulation of ROS, which deteriorates injured nerve regrowth. FGF21 possess the strong antioxidant potential to inhibit ROS production and protect SCs from apoptosis.

Oxidative stress is regulated by various intracellular signalling cascades, including ERK/Nrf‐2, PI3 K/AKT, and JAK/STAT pathways.^63^, ^64^ Among them, ERK/Nrf‐2 signalling is regarded as the pivotal molecular regulatory mechanism for combatting oxidative stress‐induced neuronal damage.^51^, ^65^, ^66^ Nrf‐2 is a key anti‐oxidant defender that cooperates with the antioxidant response element (ARE) to maintain normal oxidative levels.^67^ Mounting evidences showed that a series of Nrf‐2‐medicated antioxidant processes were involved in the upstream of MEK/ERK regulating.^68^, ^69^ For instance, acetyl‐L‐carnitine effectively protected hippocampal neurons from oxidative damage and mitochondrial dysfunction mainly through the ERK/Nrf‐2 pathway.^70^ There is increasing evidence manifested that ERK could activate the downstream transcription factor of Nrf‐2 to promote SCs proliferation and nerve repair.^30^, ^31^ Furthermore, the ERK/Nrf‐2‐mediated mechanism also resisted diabetic neuropathy induced‐oxidative damage.^32^ However, there is no clear evidence that confirmed whether FGF21‐medicated anti‐oxidative enhancing was closely related to ERK/Nrf‐2 signalling after PNI. In the present work, we found that FGF21 treating prominently upregulated phosphorylation of ERK and activated Nrf‐2 signalling pathway when compared with the PNI rats (Figure 4). These results were also confirmed in the cell lever (Figure 6). Additionally, U0126 markedly reversed the effect of FGF21 on ERK phosphorylation and Nrf‐2 activation. Taken together, these findings suggest that FGF21 enhanced antioxidant capacity to promote remyelination is probably through activating the ERK/Nrf‐2 pathway.

Autophagy is tightly linked to PNI.^71^, ^72^ Abnormal autophagic activity contributes to neuronal nonapoptotic cell death, namely “autophagic cell death”, which can be mostly reflected by the ratio of Bax and Bcl‐2.^73^, ^74^ Previous studies have demonstrated that autophagic cell death could be induced by oxidative stress.^75^ Upregulating the autophagy lever caused by oxidative damage is detrimental to neural survival and proliferation.^76^, ^77^ Suppression of excessive autophagic activation through pharmacological or genetic methods may be a novel target to reduce reactive zinc‐induced neurons and astrocytes death and alleviate the brain infarction after ischaemia injury.^78^, ^79^ These findings support the possibility that correcting autophagic dysfunction may be a potential therapeutic strategy for repairing nerve injury. In the current research, we found out the autophagy lever was excessive activation both in vivo and in vitro, which was significantly reversed after FGF21 or 3‐MA administration (Figures 5 and 7). Similar results also appeared on the ratio of Bax/Bcl‐2. The evidence suggests that abnormal autophagy activation may bring about neuronal death, which is harmful to the injured nerve regeneration. However, FGF21 is able to inhibit autophagic cell death to promote neuronal regrowth and remyelination after PNI.

In conclusion, we firstly demonstrated that exogenous FGF21 administration facilitated SCs proliferation, nerve remyelination, and functional recovery after PNI. Furthermore, this beneficial effect of FGF21 on the injured nerve and restoration, and SCs survival is likely related to suppress excessive oxidative stress‐induced cell apoptosis via the ERK/Nrf‐2 signalling pathway and autophagic cell death (Figure S2). However, we did not study the specific relationship between anti‐oxidative effect and anti‐autophagic effect regulated by FGF21. In this study, we demonstrated that FGF21 firstly promoted the expression of antioxidant enzymes when suffering from oxidative damage. Thereafter, FGF21 suppresses the excessively autophagy activated. Moreover, the extent of antioxidant capacity is higher than the inhibition effect of autophagy (Figure S1). All in all, our results suggest FGF21 administration may be a potential useful therapeutic drug for repairing PNI.

## CONFLICT OF INTERESTS

The authors declare that they have no conflicts of interest.

## References

[1] Ichihara S , Inada Y , Nakamura T . Artificial nerve tubes and their application for repair of peripheral nerve injury: an update of current concepts. Injury. 2008;39(Suppl 4):29. 18804584 10.1016/j.injury.2008.08.029

[2] Noble J , Munro CA , Prasad VS , Midha R . Analysis of upper and lower extremity peripheral nerve injuries in a population of patients with multiple injuries. J Trauma. 1998;45:116‐122. 9680023 10.1097/00005373-199807000-00025

[3] Robinson LR . Traumatic injury to peripheral nerves. Muscle Nerve. 2000;23:863‐873. 10842261 10.1002/(sici)1097-4598(200006)23:6<863::aid-mus4>3.0.co;2-0

[4] Chen P , Piao X , Bonaldo P . Role of macrophages in Wallerian degeneration and axonal regeneration after peripheral nerve injury. Acta Neuropathol. 2015;130:605‐618. 26419777 10.1007/s00401-015-1482-4

[5] Evans GR . Challenges to nerve regeneration. Semin Surg Oncol. 2000;19:312‐318. 11135488 10.1002/1098-2388(200010/11)19:3<312::aid-ssu13>3.0.co;2-m

[6] Xavier AM , Serafim KG , Higashi DT , et al. Simvastatin improves morphological and functional recovery of sciatic nerve injury in Wistar rats. Injury. 2012;43:284‐289. 21684542 10.1016/j.injury.2011.05.036

[7] Jiang X , Ma J , Wei Q , et al. Effect of frankincense extract on nerve recovery in the rat sciatic nerve damage model. Evid Based Complement Alternat Med. 2016;2016:3617216. 27143985 10.1155/2016/3617216 PMC4842080

[8] Allodi I , Mecollari V , Gonzalez‐Perez F , et al. Schwann cells transduced with a lentiviral vector encoding Fgf‐2 promote motor neuron regeneration following sciatic nerve injury. Glia. 2014;62:1736‐1746. 24989458 10.1002/glia.22712

[9] Itoh N . Hormone‐like (endocrine) Fgfs: their evolutionary history and roles in development, metabolism, and disease. Cell Tissue Res. 2010;342:1‐11. 20730630 10.1007/s00441-010-1024-2 PMC2948652

[10] Kelleher FC , O'Sullivan H , Smyth E , McDermott R , Viterbo A . Fibroblast growth factor receptors, developmental corruption and malignant disease. Carcinogenesis. 2013;34:2198‐2205. 23880303 10.1093/carcin/bgt254

[11] Yu Y , Bai F , Liu Y , et al. Fibroblast growth factor (FGF21) protects mouse liver against D‐galactose‐induced oxidative stress and apoptosis via activating Nrf2 and PI3K/Akt pathways. Mol Cell Biochem. 2015;403:287‐299. 25701356 10.1007/s11010-015-2358-6

[12] Cheng Y , Zhang J , Guo W , et al. Up‐regulation of Nrf2 is involved in FGF21‐mediated fenofibrate protection against type 1 diabetic nephropathy. Free Radic Biol Med. 2016;93:94‐109. 26849944 10.1016/j.freeradbiomed.2016.02.002 PMC7446394

[13] Gomez‐Samano MA , Grajales‐Gomez M , Zuarth‐Vazquez JM , et al. Fibroblast growth factor 21 and its novel association with oxidative stress. Redox Biol. 2017;11:335‐341. 28039838 10.1016/j.redox.2016.12.024 PMC5200873

[14] Omar BA , Andersen B , Hald J , Raun K , Nishimura E , Ahren B . Fibroblast growth factor 21 (FGF21) and glucagon‐like peptide 1 contribute to diabetes resistance in glucagon receptor‐deficient mice. Diabetes. 2014;63:101‐110. 24062250 10.2337/db13-0710

[15] Kim KH , Lee MS . FGF21 as a mediator of adaptive responses to stress and metabolic benefits of anti‐diabetic drugs. J Endocrinol. 2015;226:R1‐R16. 26116622 10.1530/JOE-15-0160

[16] Li Q , Zhang Y , Ding D , et al. Association between serum fibroblast growth factor 21 and mortality among patients with coronary artery disease. J Clin Endocrinol Metabol. 2016;101:4886‐4894. 10.1210/jc.2016-2308 27662438

[17] Chen J , Hu J , Liu H , et al. FGF21 protects the blood‐brain barrier by upregulating PPARgamma via FGFR1/beta‐Klotho following traumatic brain injury. J Neurotrauma 2018;35(17):2091‐2103. 29648978 10.1089/neu.2017.5271

[18] Planavila A , Redondo‐Angulo I , Ribas F , et al. Fibroblast growth factor 21 protects the heart from oxidative stress. Cardiovasc Res. 2015;106:19‐31. 25538153 10.1093/cvr/cvu263

[19] Yu Y , Bai F , Wang W , et al. Fibroblast growth factor 21 protects mouse brain against D‐galactose induced aging via suppression of oxidative stress response and advanced glycation end products formation. Pharmacol Biochem Behav. 2015;133:122‐131. 25871519 10.1016/j.pbb.2015.03.020

[20] Leng Y , Wang Z , Tsai LK , et al. FGF‐21, a novel metabolic regulator, has a robust neuroprotective role and is markedly elevated in neurons by mood stabilizers. Mol Psychiatry. 2015;20:215‐223. 24468826 10.1038/mp.2013.192 PMC4113566

[21] Kuroda M , Muramatsu R , Maedera N , et al. Peripherally derived FGF21 promotes remyelination in the central nervous system. J Clin Investig. 2017;127:3496‐3509. 28825598 10.1172/JCI94337 PMC5669554

[22] Jain S , Webster TJ , Sharma A , Basu B . Intracellular reactive oxidative stress, cell proliferation and apoptosis of Schwann cells on carbon nanofibrous substrates. Biomaterials. 2013;34:4891‐4901. 23570716 10.1016/j.biomaterials.2013.03.055

[23] Hsu CC , Huang HC , Wu PT , Tai TW , Jou IM . Sesame oil improves functional recovery by attenuating nerve oxidative stress in a mouse model of acute peripheral nerve injury: role of Nrf‐2. J Nutr Biochem. 2016;38:102‐106. 27732910 10.1016/j.jnutbio.2016.09.003

[24] Ress AM , Babovic S , Angel MF , Im MJ , Dellon AL , Manson PN . Free radical damage in acute nerve compression. Ann Plast Surg. 1995;34:388‐395. 7793785 10.1097/00000637-199504000-00009

[25] Do MT , Kim HG , Khanal T , et al. Metformin inhibits heme oxygenase‐1 expression in cancer cells through inactivation of Raf‐ERK‐Nrf2 signaling and AMPK‐independent pathways. Toxicol Appl Pharmacol. 2013;271:229‐238. 23707609 10.1016/j.taap.2013.05.010

[26] Wilcox CS , Pearlman A . Chemistry and antihypertensive effects of tempol and other nitroxides. Pharmacol Rev. 2008;60:418‐469. 19112152 10.1124/pr.108.000240 PMC2739999

[27] Mohagheghi F , Khalaj L , Ahmadiani A , Rahmani B . Gemfibrozil pretreatment affecting antioxidant defense system and inflammatory, but not Nrf‐2 signaling pathways resulted in female neuroprotection and male neurotoxicity in the rat models of global cerebral ischemia‐reperfusion. Neurotox Res. 2013;23:225‐237. 22773136 10.1007/s12640-012-9338-3

[28] Zhang DD . Mechanistic studies of the Nrf2‐Keap1 signaling pathway. Drug Metab Rev. 2006;38:769‐789. 17145701 10.1080/03602530600971974

[29] Junttila MR , Li SP , Westermarck J . Phosphatase‐mediated crosstalk between MAPK signaling pathways in the regulation of cell survival. FASEB J. 2008;22:954‐965. 18039929 10.1096/fj.06-7859rev

[30] Harrisingh MC , Perez‐Nadales E , Parkinson DB , Malcolm DS , Mudge AW , Lloyd AC . The Ras/Raf/ERK signalling pathway drives Schwann cell dedifferentiation. EMBO J. 2004;23:3061‐3071. 15241478 10.1038/sj.emboj.7600309 PMC514926

[31] Newbern JM , Snider WD . Bers‐ERK Schwann cells coordinate nerve regeneration. Neuron. 2012;73:623‐626. 22365537 10.1016/j.neuron.2012.02.002

[32] Wang HQ , Xu YX , Zhu CQ . Upregulation of heme oxygenase‐1 by acteoside through ERK and PI3 K/Akt pathway confer neuroprotection against beta‐amyloid‐induced neurotoxicity. Neurotox Res. 2012;21:368‐378. 22147269 10.1007/s12640-011-9292-5

[33] Castillo K , Valenzuela V , Matus S , et al. Measurement of autophagy flux in the nervous system in vivo. Cell Death Dis. 2013;4:e917. 24232093 10.1038/cddis.2013.421 PMC3847309

[34] Liu Y , Levine B . Autosis and autophagic cell death: the dark side of autophagy. Cell Death Differ. 2015;22:367‐376. 25257169 10.1038/cdd.2014.143 PMC4326571

[35] Descloux C , Ginet V , Clarke PG , Puyal J , Truttmann AC . Neuronal death after perinatal cerebral hypoxia‐ischemia: focus on autophagy‐mediated cell death. Int J Dev Neurosci. 2015;45:75‐85. 26225751 10.1016/j.ijdevneu.2015.06.008

[36] Sarkar C , Zhao Z , Aungst S , Sabirzhanov B , Faden AI , Lipinski MM . Impaired autophagy flux is associated with neuronal cell death after traumatic brain injury. Autophagy. 2014;10:2208‐2222. 25484084 10.4161/15548627.2014.981787 PMC4502690

[37] Yin DH , Liang XC , Zhao LI , et al. Jinmaitong decreases sciatic nerve DNA oxidative damage and apoptosis in a streptozotocin‐induced diabetic rat model. Exp Ther Med. 2015;10:778‐786. 26622393 10.3892/etm.2015.2543 PMC4509113

[38] Huang K , Chen Y , Zhang R , et al. Honokiol induces apoptosis and autophagy via the ROS/ERK1/2 signaling pathway in human osteosarcoma cells in vitro and in vivo. Cell Death Dis. 2018;9:157. 29410403 10.1038/s41419-017-0166-5 PMC5833587

[39] Shi G , Shi J , Liu K , et al. Increased miR‐195 aggravates neuropathic pain by inhibiting autophagy following peripheral nerve injury. Glia. 2013;61:504‐512. 23361941 10.1002/glia.22451

[40] Li R , Wu J , Lin Z , et al. Single injection of a novel nerve growth factor coacervate improves structural and functional regeneration after sciatic nerve injury in adult rats. Exp Neurol. 2017;288:1‐10. 27983992 10.1016/j.expneurol.2016.10.015

[41] Bain JR , Mackinnon SE , Hudson AR , Falk RE , Falk JA , Hunter DA . The peripheral nerve allograft: an assessment of regeneration across nerve allografts in rats immunosuppressed with cyclosporin A. Plast Reconstr Surg. 1988;82:1052‐1066. 3264409

[42] Li R , Li Y , Wu Y , et al. Heparin‐poloxamer thermosensitive hydrogel loaded with bFGF and NGF enhances peripheral nerve regeneration in diabetic rats. Biomaterials. 2018;168:24‐37. 29609091 10.1016/j.biomaterials.2018.03.044 PMC5935004

[43] Li R , Wu Y , Zou S , et al. NGF attenuates high glucose‐induced er stress, preventing schwann cell apoptosis by activating the PI3K/Akt/GSK3beta and ERK1/2 pathways. Neurochem Res. 2017;42(11):3005‐3018. 28762104 10.1007/s11064-017-2333-6

[44] Gaudet AD , Popovich PG , Ramer MS . Wallerian degeneration: gaining perspective on inflammatory events after peripheral nerve injury. J Neuroinflammation. 2011;8:110. 21878126 10.1186/1742-2094-8-110 PMC3180276

[45] Fu SY , Gordon T . Contributing factors to poor functional recovery after delayed nerve repair: prolonged denervation. J Neurosci. 1995;15:3886‐3895. 7751953 10.1523/JNEUROSCI.15-05-03886.1995 PMC6578254

[46] Ravera S , Bartolucci M , Cuccarolo P , et al. Oxidative stress in myelin sheath: the other face of the extramitochondrial oxidative phosphorylation ability. Free Radical Res. 2015;49:1156‐1164. 25971447 10.3109/10715762.2015.1050962

[47] Monk KR , Feltri ML , Taveggia C . New insights on Schwann cell development. Glia. 2015;63:1376‐1393. 25921593 10.1002/glia.22852 PMC4470834

[48] Jessen KR , Mirsky R . The origin and development of glial cells in peripheral nerves. Nat Rev Neurosci. 2005;6:671‐682. 16136171 10.1038/nrn1746

[49] Zhao Z , Li X , Li Q . Curcumin accelerates the repair of sciatic nerve injury in rats through reducing Schwann cells apoptosis and promoting myelinization. Biomed Pharmacother 2017;92:1103‐1110. 28622711 10.1016/j.biopha.2017.05.099

[50] Lisak RP , Nedelkoska L , Benjamins JA . Effects of dextromethorphan on glial cell function: proliferation, maturation, and protection from cytotoxic molecules. Glia. 2014;62:751‐762. 24526455 10.1002/glia.22639

[51] Cheung KL , Lee JH , Shu L , Kim JH , Sacks DB , Kong AN . The Ras GTPase‐activating‐like protein IQGAP1 mediates Nrf2 protein activation via the mitogen‐activated protein kinase/extracellular signal‐regulated kinase (ERK) kinase (MEK)‐ERK pathway. J Biol Chem. 2013;288:22378‐22386. 23788642 10.1074/jbc.M112.444182 PMC3829328

[52] Zhang J , Cai Q , Jiang M , et al. Mesencephalic astrocyte‐derived neurotrophic factor alleviated 6‐OHDA‐induced cell damage via ROS‐AMPK/mTOR mediated autophagic inhibition. Exp Gerontol. 2017;89:45‐56. 28099881 10.1016/j.exger.2017.01.010

[53] Li H , Zhang J , Jia W . Fibroblast growth factor 21: a novel metabolic regulator from pharmacology to physiology. Front Med. 2013;7:25‐30. 23358894 10.1007/s11684-013-0244-8

[54] Chen JK , Yao LL , Jenq CB . Mitogenic response of rat Schwann cells to fibroblast growth factors is potentiated by increased intracellular cyclic AMP levels. J Neurosci Res. 1991;30:321‐327. 1665866 10.1002/jnr.490300207

[55] Gavrilovic J , Brennan A , Mirsky R , Jessen KR . Fibroblast growth factors and insulin growth factors combine to promote survival of rat Schwann cell precursors without induction of DNA synthesis. Eur J Neuorsci. 1995;7:77‐85. 10.1111/j.1460-9568.1995.tb01022.x 7711939

[56] Fu X , Shen Z , Chen Y , et al. Randomised placebo‐controlled trial of use of topical recombinant bovine basic fibroblast growth factor for second‐degree burns. Lancet. 1998;352:1661‐1664. 9853438 10.1016/S0140-6736(98)01260-4

[57] Yu Y , He J , Li S , et al. Fibroblast growth factor 21 (FGF21) inhibits macrophage‐mediated inflammation by activating Nrf2 and suppressing the NF‐kappaB signaling pathway. Int Immunopharmacol. 2016;38:144‐152. 27276443 10.1016/j.intimp.2016.05.026

[58] Namgung U . The role of Schwann cell‐axon interaction in peripheral nerve regeneration. Cells Tissues Organs. 2014;200:6‐12. 25765065 10.1159/000370324

[59] Schmeichel AM , Schmelzer JD , Low PA . Oxidative injury and apoptosis of dorsal root ganglion neurons in chronic experimental diabetic neuropathy. Diabetes. 2003;52:165‐171. 12502508 10.2337/diabetes.52.1.165

[60] Areti A , Yerra VG , Naidu V , Kumar A . Oxidative stress and nerve damage: role in chemotherapy induced peripheral neuropathy. Redox Biol. 2014;2:289‐295. 24494204 10.1016/j.redox.2014.01.006 PMC3909836

[61] Kaya Y , Savas K , Sarikcioglu L , Yaras N , Angelov DN . Melatonin leads to axonal regeneration, reduction in oxidative stress, and improved functional recovery following sciatic nerve injury. Curr Neurovasc Res. 2015;12:53‐62. 25557375 10.2174/1567202612666150102151900

[62] Dasuri K , Zhang L , Keller JN . Oxidative stress, neurodegeneration, and the balance of protein degradation and protein synthesis. Free Radic Biol Med. 2013;62:170‐185. 23000246 10.1016/j.freeradbiomed.2012.09.016

[63] Liu H , Li X , Qin F , Huang K . Selenium suppresses oxidative‐stress‐enhanced vascular smooth muscle cell calcification by inhibiting the activation of the PI3K/AKT and ERK signaling pathways and endoplasmic reticulum stress. J Biol Inorg Chem. 2014;19:375‐388. 24390545 10.1007/s00775-013-1078-1

[64] Park SK , Dahmer MK , Quasney MW . MAPK and JAK‐STAT signaling pathways are involved in the oxidative stress‐induced decrease in expression of surfactant protein genes. Cell Physiol Biochem. 2012;30:334‐346. 22739240 10.1159/000339068

[65] Wakabayashi N , Slocum SL , Skoko JJ , Shin S , Kensler TW . When NRF2 talks, who's listening? Antioxid Redox Signal. 2010;13:1649‐1663. 20367496 10.1089/ars.2010.3216 PMC2966480

[66] Hayes JD , McMahon M , Chowdhry S , Dinkova‐Kostova AT . Cancer chemoprevention mechanisms mediated through the Keap1‐Nrf2 pathway. Antioxid Redox Signal. 2010;13:1713‐1748. 20446772 10.1089/ars.2010.3221

[67] Giudice A , Arra C , Turco MC . Review of molecular mechanisms involved in the activation of the Nrf2‐ARE signaling pathway by chemopreventive agents. Methods Mol Biol. 2010;647:37‐74. 20694660 10.1007/978-1-60761-738-9_3

[68] Kobayashi M , Yamamoto M . Molecular mechanisms activating the Nrf2‐Keap1 pathway of antioxidant gene regulation. Antioxid Redox Signal. 2005;7:385‐394. 15706085 10.1089/ars.2005.7.385

[69] Andreadi CK , Howells LM , Atherfold PA , Manson MM . Involvement of Nrf2, p38, B‐Raf, and nuclear factor‐kappaB, but not phosphatidylinositol 3‐kinase, in induction of hemeoxygenase‐1 by dietary polyphenols. Mol Pharmacol. 2006;69:1033‐1040. 16354769 10.1124/mol.105.018374

[70] Hota KB , Hota SK , Chaurasia OP , Singh SB . Acetyl‐L‐carnitine‐mediated neuroprotection during hypoxia is attributed to ERK1/2‐Nrf2‐regulated mitochondrial biosynthesis. Hippocampus. 2012;22:723‐736. 21542052 10.1002/hipo.20934

[71] Jang SY , Shin YK , Park SY , et al. Autophagic myelin destruction by Schwann cells during Wallerian degeneration and segmental demyelination. Glia. 2016;64:730‐742. 26712109 10.1002/glia.22957

[72] Huang HC , Chen L , Zhang HX , et al. Autophagy promotes peripheral nerve regeneration and motor recovery following sciatic nerve crush injury in rats. J Mol Neurosci. 2016;58:416‐423. 26738732 10.1007/s12031-015-0672-9 PMC4829621

[73] Chang MY , Sun W , Ochiai W , et al. Bcl‐XL/Bax proteins direct the fate of embryonic cortical precursor cells. Mol Cell Biol. 2007;27:4293‐4305. 17438128 10.1128/MCB.00031-07 PMC1900045

[74] Kluck RM , Esposti MD , Perkins G , et al. The pro‐apoptotic proteins, Bid and Bax, cause a limited permeabilization of the mitochondrial outer membrane that is enhanced by cytosol. J Cell Biol. 1999;147:809‐822. 10562282 10.1083/jcb.147.4.809 PMC2156156

[75] Navarro‐Yepes J , Burns M , Anandhan A , et al. Oxidative stress, redox signaling, and autophagy: cell death versus survival. Antioxid Redox Signal. 2014;21:66‐85. 24483238 10.1089/ars.2014.5837 PMC4048575

[76] Xu Y , Tian Y , Tian Y , Li X , Zhao P . Autophagy activation involved in hypoxic‐ischemic brain injury induces cognitive and memory impairment in neonatal rats. J Neurochem. 2016;139:795‐805. 27659442 10.1111/jnc.13851

[77] Cherra SJ 3rd , Chu CT . Autophagy in neuroprotection and neurodegeneration: a question of balance. Future Neurol. 2008;3:309‐323. 18806889 10.2217/14796708.3.3.309 PMC2544613

[78] Lee SJ , Koh JY . Roles of zinc and metallothionein‐3 in oxidative stress‐induced lysosomal dysfunction, cell death, and autophagy in neurons and astrocytes. Mol Brain. 2010;3:30. 20974010 10.1186/1756-6606-3-30 PMC2988061

[79] Zhang L , Niu W , He Z , et al. Autophagy suppression by exercise pretreatment and p38 inhibition is neuroprotective in cerebral ischemia. Brain Res. 2014;1587:127‐132. 25192645 10.1016/j.brainres.2014.08.067

