# Supplementary figures and images for: Fibroblast growth factor 21 facilitates peripheral nerve regeneration through suppressing oxidative damage and autophagic cell death
Source: J Cell Mol Med. 2018 Nov 18;23(1):497–511. doi: 10.1111/jcmm.13952 (PMC6307793; doi:10.1111/jcmm.13952)

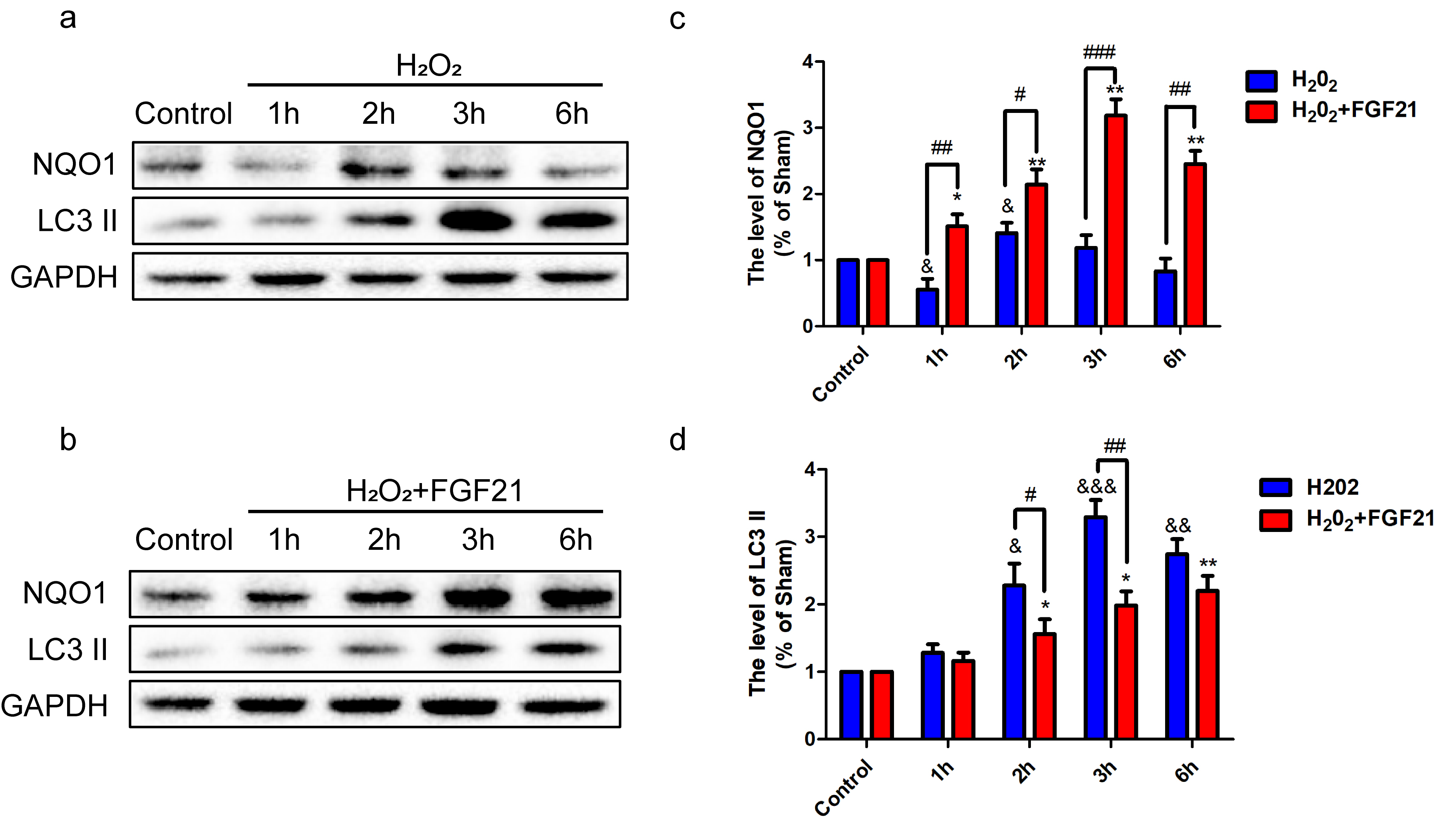

Supplement: Supplementary file 1 [file JCMM-23-497-s001.tif]

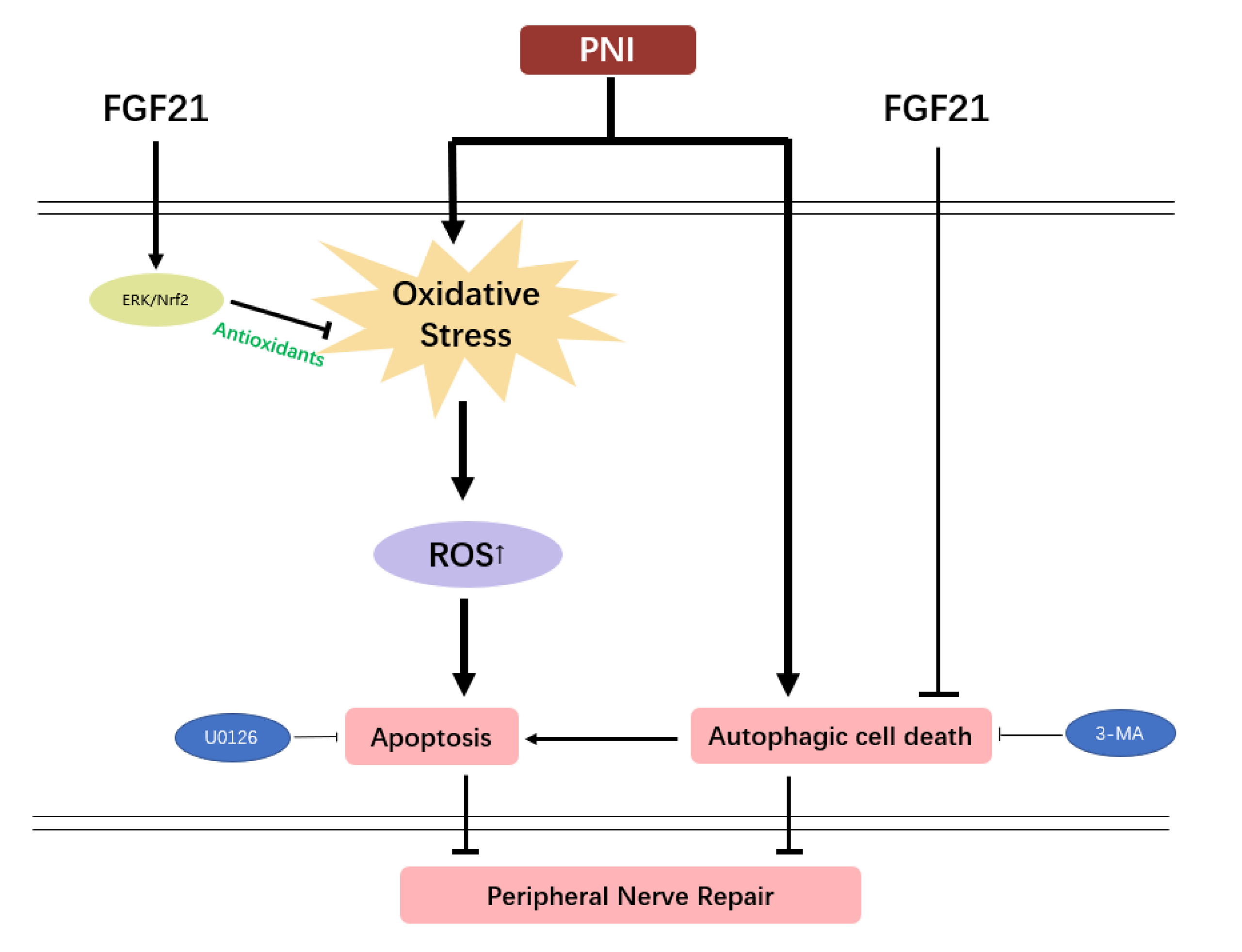

Supplement: Supplementary file 2 [file JCMM-23-497-s002.tif]
